# The Intellectual Structure of Research on Rural-to-Urban Migrants: A Bibliometric Analysis

**DOI:** 10.3390/ijerph19159729

**Published:** 2022-08-07

**Authors:** Huichen Gao, Shijuan Wang

**Affiliations:** 1 School of Economics, Zhongnan University of Economics and Law, Wuhan 430073, China; 2 Faculty of Artificial Intelligence Education, Central China Normal University, Wuhan 430079, China

**Keywords:** rural-to-urban migrant, bibliometric analysis, performance analysis, science mapping, network analysis

## Abstract

As noted in the United Nations’ Sustainable Development Goals 2030 agenda, sustainable cities “without leaving anyone behind” should take into consideration migrant groups, which may play only a marginal role but may be at the root of potential social conflicts. This study thereby promotes cross-disciplinary explorations of knowing and understanding the rural-to-urban internal migrants against the background of rapid urbanization. This study conducted a bibliometric analysis based on 2788 English language articles obtained from the Web of Science Core Collection database. As China’s unique Hukou system highlights the divide between rural migrants and urban dwellers, migrant studies have extended to a diverse range of interests. We underlined the most productive sources and authors in this area and identified networks of collaboration among countries and institutions. Furthermore, we found trends in research themes and topics and research clusters through keyword-based analysis techniques. The results provide a rich source of information on the intellectual structure of the chosen domain of rural-to-urban migrants.

## 1. Introduction

In this era of accelerating globalization and rapid urbanization, cities and metropolitan areas play an essential role in pooling resources and providing opportunities for people to prosper both economically and socially. With a global share of the world population that is projected to reach 68% in 2050, urbanized areas are experiencing challenges in meeting the growing demand for public services while maintaining a sustainable environment for present and future generations [1]. Yet massive influxes of capital and preferential policies have created uneven economic development between rural and urban areas (as seen in China’s dual economy), helping drive migration to cities [2,3,4]. As noted in the United Nations’ Sustainable Development Goals (SDG) 2030 agenda, sustainable cities “without leaving anyone behind” should take into consideration migrant groups, which may play only a marginal role but may be at the root of potential social conflicts [5]. As a cross-cutting issue on the SDG 2030 agenda, migration is attracting the attention of researchers from multiple disciplines, and it poses novel challenges to the public and policymakers at both the national and international levels [6]. COVID-19 has sounded the alarm and drawn additional attention to unmet health needs within migrant communities [7]. Our research provides a lens to examine a particular group of migrants, namely, rural-to-urban internal migrants. Previous studies on migrant groups in urban wards cover a broad spectrum of themes and topics across disciplines, providing a vivid but fractional profile of this group of people [8,9]. Since the application of bibliometric methods in migration research is relatively new, we consider that it might be a good time to conduct an analysis with newly developed tools such as VOSviewer to interpret the intellectual structures of research on rural-to-urban migrants. This study thereby promotes cross-disciplinary explorations of knowing and understanding the migrant population against the background of rapid urbanization.

The article presents a bibliometric analysis aimed at sketching the outline of knowledge related to rural-to-urban migration at the initial stage (Section 3.1 and Section 3.2). The social structure and collaboration patterns are then identified using network analysis based on co-authorship data (Section 3.3). Then, bibliometric analysis results allowed another analysis to be conducted to examine the coupling network of the documents and the co-citation network of references, which aids in mapping current areas of focus and landmark studies from the past (Section 3.4). This is followed by a keywords network analysis in Section 3.5, which briefly describes keyword frequency and three distinct subsections: keyword co-occurrence network analysis, thematic analysis, and conceptual structure analysis. Finally, the findings and policy implications are summarized.

## 2. Research Methodology

Researchers have applied this comprehensive science mapping approach to multiple domains, such as rural depopulation [10] and urban agriculture [11] since Aria and Cuccurullo [12] published the bibliometrix R-package. This one-of-a-kind open-source tool developed in R language offers a wide range of statistical and graphical techniques and a user-friendly web-interface application called Biblioshiny. As demonstrated by Rogers et al. [13], data collected for bibliometric analysis are typically massive. A bibliometric analysis of 200 papers appears to be a reasonable threshold to employ a bibliometric analysis. The bibliometric analysis applied in this research is conducted using the bibliometrix package (version 3.2.1), built in R (version 4.1.3). VOSviewer (version 1.6.18) is a well-developed visualization tool for building and viewing bibliometric networks [14]. When a “net” object is created in the R Programming Environment, VOSviewer functions using internal R routines.

The bibliographic data for this study were collected on 3 June 2022, from the Web of Science (WOS) Core Collection database, including indices of SCIE, SSCI, AHCI, ESCI, CPCI, BKCI and CCR&IC, provided by Clarivate within the indexed timespan from 1 January 2004 to 3 June 2022. Using the topic keyword “rural urban migrant” (the topic keywords are generated by Clarivate from the title, abstract, author keywords, and Keywords Plus) and filtering by English language and the document type “article,” we obtained a total of 2790 publications in the first stage. After removing duplicates, 2788 papers remained. Each document’s full record and cited references are exported for the bibliometric analysis.

## 3. Results and Discussion

### 3.1. Descriptive Bibliometric Analysis

**Figure 1:**
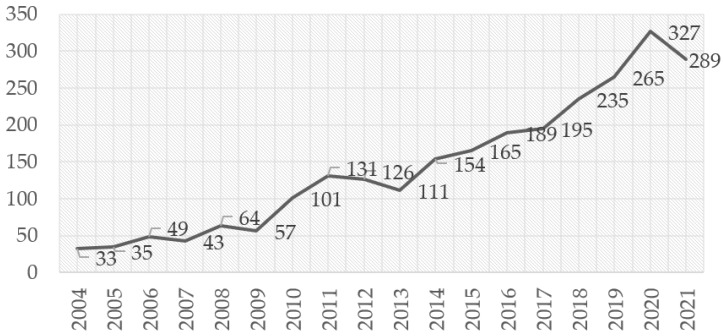
Number of articles published on the topic of rural-to-urban migrants from 2004 to 2021.

As shown in Table 1, since 2004, 2788 articles have been published in this specific field in 1006 venues. Excluding 2022, the annual growth rate over this period is 13.61% (Figure 1). The total citations per article reached a peak of 68.1 in 2007, and the total number of references was 98860. Overall, 5626 author keywords and 3624 Keywords Plus were generated. As shown in Figure 1, the increase in publications since 2010 is noticeable, despite a slight decline in the years 2012–2013.

**Figure 2:**
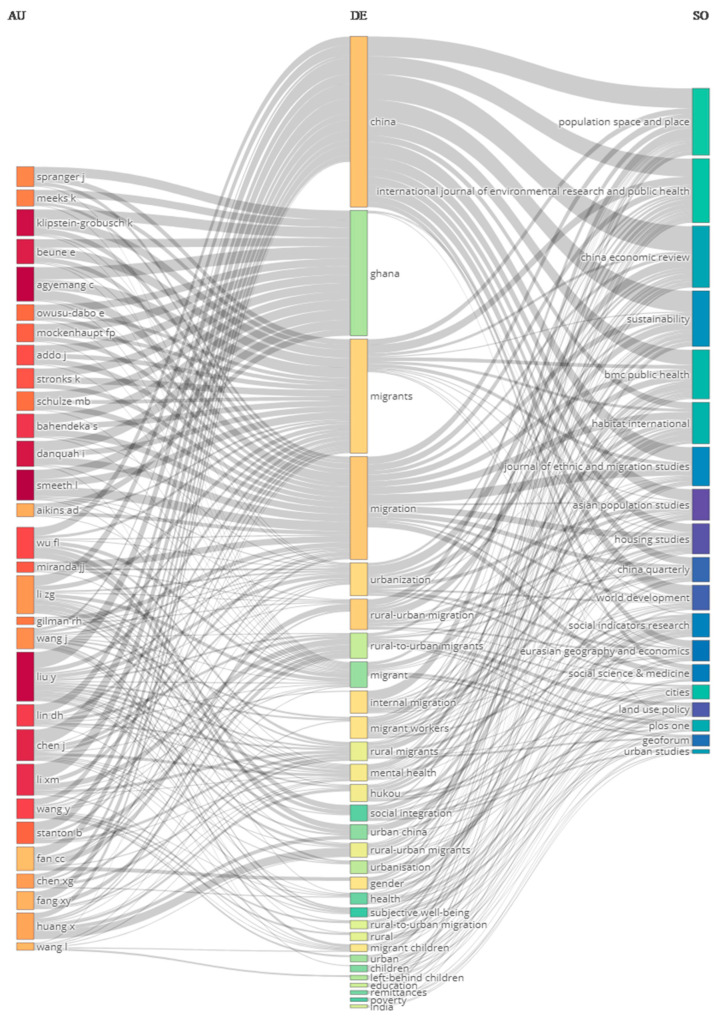
A Sankey diagram showing authors (AU), author keywords (DE), and sources (SO).

Statistical analysis was conducted based on Lotka’s law, which measures the frequency distribution of scientific productivity [15]. Simply put, Lotka’s Law states that there is a consistent ratio between the number of authors publishing a certain number of papers and the number of authors who publish a single one. The results show that the distribution of author frequency and number of publications follows Lotka’s Law (R² = 0.920, *p*-value = 0.006). In all, 4546 authors contributed to one article each, accounting for 78.9% of all authors; of the remainder, 641 authors contributed to two articles (11.1%), 239 to three (4.1%), 113 to four (2.0%), and 222 to five or more articles (3.9%) regarding the topic addressed by this study. Figure 2 shows a Sankey diagram that presents a visualization of the connections between the main items of three fields, namely, authors, author keywords, and sources. We find that “China” and “Ghana” are two popular areas of research interest. A detailed description is given next.

### 3.2. Top Sources

**Figure 3:**
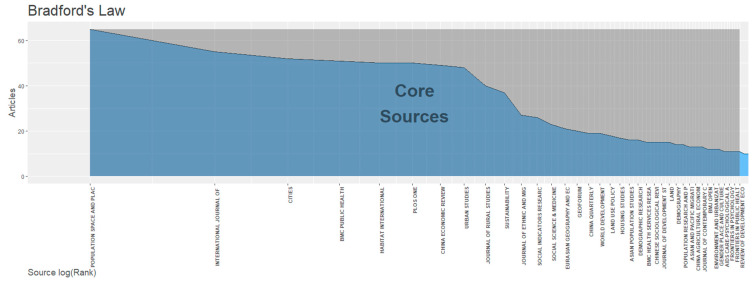
Core sources on the topic of rural-to-urban migrants (1 January 2004–3 June 2022).

A Bradford analysis was conducted (Figure 3), and from 1006 sources, 37 core scientific journals were identified, marked as Zone 1 [16]. Zones 2 and 3 contain 203 and 766 sources, respectively. Each zone carries an equal number of citations.

The top 10 high-yield journals from Zone 1 are listed in Table 2, accounting for 17.8% of the total number of publications and 22% of the total citations. These are primarily international interdisciplinary journals covering multiple topics, such as occupational health and urban planning. H-indexes are not used to compare the sources in our analysis because different disciplines’ citation traditions and methods vary.

### 3.3. Social Structure

#### 3.3.1. Active Countries

Papers have been published by scholars from 76 countries (counting only the corresponding author’s country) since 2004, and this number reaches 93 if co-authors are taken into account. Table 3 shows the countries with the highest output based on the frequency distribution of the corresponding author’s affiliation country. China has the leading position, with 1035 papers, accounting for 37.4% of all published papers, followed by the United States (528 papers, 19.5%) and the United Kingdom (241 papers, 8.7%). The United Kingdom and the United States rank second (24.78) and third (23.64), respectively, in terms of average citations per article, while India has a relatively low frequency (7.35).

#### 3.3.2. Active Institutions

**Figure 4:**
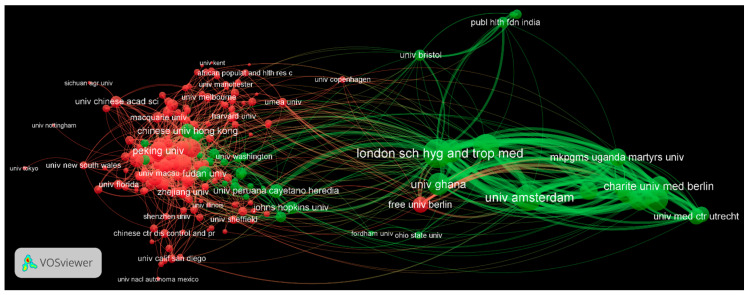
Network of institutional cooperation.

Since 2004, 1973 institutions have engaged in the study of rural-to-urban migrants. Table 4 lists the top 10 high-yield institutions according to the number of publications, of which eight are from China. A collaboration network analysis is performed, using the affiliations of each co-author and corresponding author. The degree of centrality, the number of relational ties an objective has in a network, is calculated to enrich the bibliometric assessment of institutional cooperation [17]. The collaboration patterns among institutions presented in Figure 4 identify two major networks in this field: roughly defined, one is based in China and one is based in Europe. It should be noted that the large number of publications originating in China (1035, 37.4% of total) makes it a dominant player within the global network.

Moreover, a closer look at papers from the London School of Hygiene and Tropical Medicine, the fifth-ranking institution of origination, shows that its research interests are mainly centered on the health issues of residents of or migrants from sub-Saharan African countries. Work involving the Research on Obesity and Diabetes among African Migrants is an example of this (e.g., [18]). The League of European Research Universities, such as the University of Amsterdam and Utrecht University, can also be found within these networks, collaborating with African institutions, e.g., the University of Ghana and the University of the Witwatersrand.

#### 3.3.3. Active Authors

**Figure 5:**
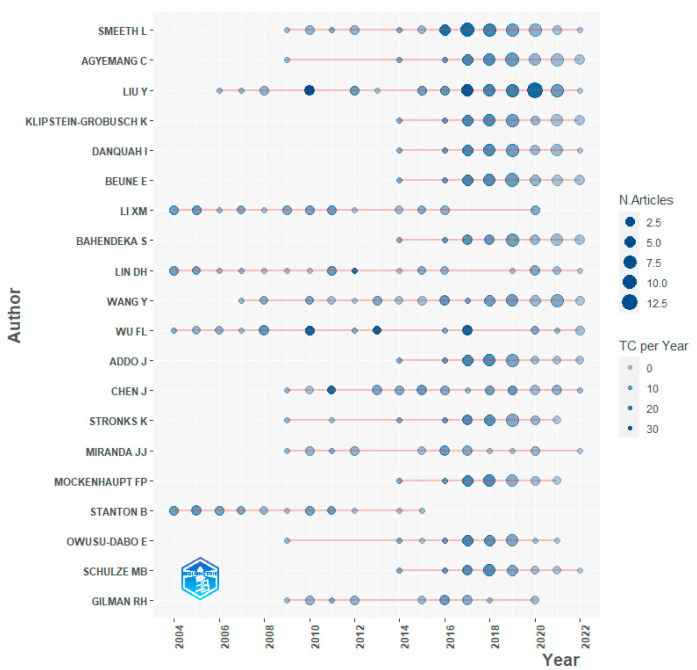
Top 20 authors’ productivity on the topic of rural-to-urban migrants (1 January 2004–3 June 2022).

Figure 5 shows 20 active authors in this field, including statistical data on the number of articles published per year by each and total citations each year. All of these authors have conducted long-term research on the topic of rural-to-urban migrants for 8–19 years. For example, Professor Liam Smeeth at the London School of Hygiene and Tropical Medicine has published papers on the topic (51 articles were identified in this study) since 2009; Professor Fulong Wu at University College London, whose interests include rural migrants in urban China, has maintained a productive research career (having published 27 articles found in this study) that spans the entire period covered by this study.

### 3.4. Most-Cited Articles

The analysis in this section uses the bibliometrix R-package to detect the historical roots of the set topic by examining each document’s bibliography [19]. The results identify references as far back as the 18th century, e.g., [20]. The historical studies uncovered by this research explore regional imbalances, migration movements in the old times, and the spread of disease that accompanies the population flow [21,22,23].

Because the term for the articles retrieved from the WOS Core Collection database for this study is after 2004, the analysis of bibliographic coupling is of more help for mapping current fronts on which research is expanding than identifying schools of thought over time [24]. Thus, a co-citation analysis is provided to improve our understanding of any paradigm shift that lies in the references [25]. In simple terms, we analyze both the retrieved documents and their references to map current areas of focus and landmark studies from the past.

Table 5 shows the top 10 works for each category. In general, these are high-value nodes connected with other nodes in the coupling network or the co-citation network. Once again, studies of contemporary China have made a significant contribution to our understanding of urbanization and migration, raising concerns about the lack of a global perspective.

### 3.5. Keywords Network Analysis

Keywords analysis is a core method in bibliometric analysis, due to the high degree of clarification and identification it provides, enabling a new approach to tracing the evolution of a discipline and determining the structure of a scientific field [45,46]. The results of the keywords network analysis are presented as follows.

#### 3.5.1. Keyword Frequency

**Figure 6:**
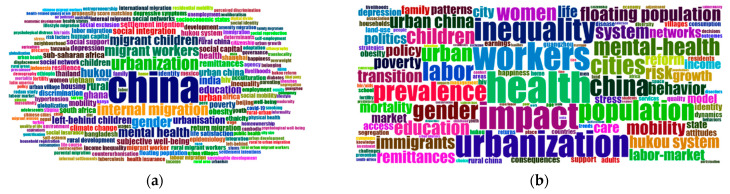
Word cloud of ranking distribution with author keywords (**a**) and Keywords Plus (**b**).

As shown in Figure 6, keyword frequency is measured and demonstrated using the word cloud technique. Given that the database was created through a topic search using “rural urban migrant” as a keyword phrase, we removed prominent high-frequency words that appear in or are closely related to this phrase (rural-to-urban, migrant, migration, etc.) to allow others to surface.

Both figures indicate where the research focus falls, although the one derived from the author’s keywords shows a higher concentration of research on China, a relatively high concentration, which is easy to understand, due to the rapid and widespread urbanization that has been taking place in China over the past couple of years. The word cloud derived from Keyword Plus indicates a diverse range of research interests, such as those regarding left-behind children, gender inequality, and mental health.

**Figure 7:**
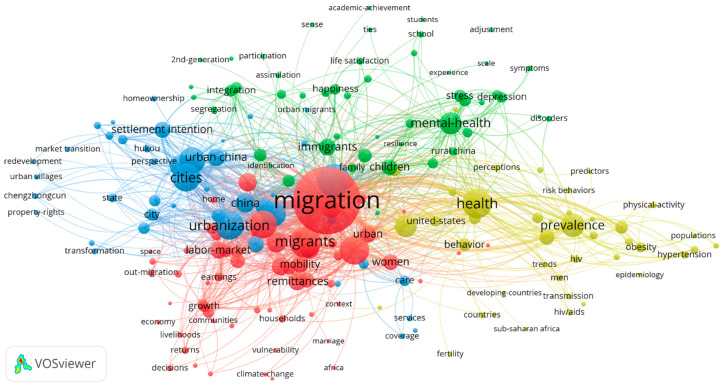
Co-occurrences of Keyword Plus on the topic of rural-to-urban migrants (1 January 2004–3 June 2022) (*n* = 200).

A keyword co-occurrence analysis (presented in Figure 7) using R and the visualization tool VOSviewer provides a co-occurrence network picture of the keyword universe and a much deeper understanding of the interaction dynamics of the field of migrant research [47,48]. Keyword Plus terms are used as inputs for the analysis because they are more broadly descriptive than author keywords [49]. Social inequality and physical and mental health are two major areas of migrant studies. A more detailed discussion of research clusters on rural-to-urban migrants is given in the following section, enhanced by a conceptual structure map.

#### 3.5.2. Trend Topics and Thematic Map

**Figure 8:**
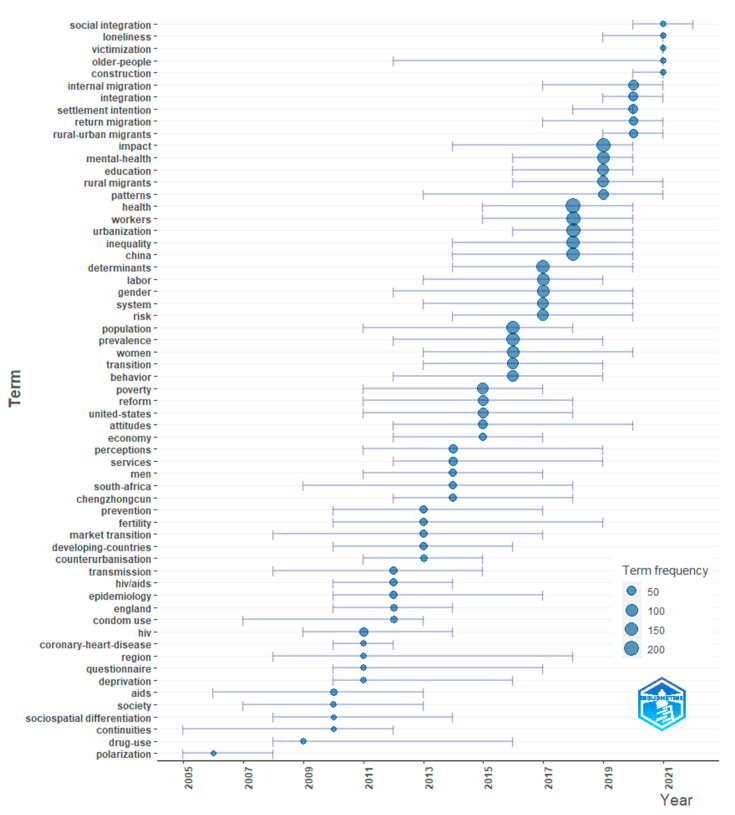
Trend topics of studies on rural-to-urban migrants (1 January 2004–3 June 2022).

An analysis of trend topics provides further information on timelines and represents a knowledge transfer between topics at different periods [50]. Figure 8 presents a profile of research trajectories in the migrant research literature, from disease prevalence as the population moves, to labor market dynamics and interactions, and to settlement intention and social integration.

Co-word analysis is performed across the entire period to detect and visualize specific themes, and bibliometric measures, such as the h-index, are used to evaluate the performance of each theme [51]. In other words, thematic evolution is examined by combining the performances of specific themes identified by co-word analysis at various subperiods. In the following step, two measures, Callon’s centrality (*c* = 10 ×$\sum e_{kh}$ with *e* the equivalence index, *k* a local keyword, and *h* a keyword from other themes) and Callon’s density (*d* = 100 $\sum \frac{e_{ij}}{w}$ with *e* the equivalence index, *i* and *j* both local keywords and *w* the number of keywords in the theme), are taken to measure the strength of external ties, that is, a theme to other themes and the internal strength of a particular theme, respectively [48,52]. Accordingly, we include a thematic analysis based on the frequency of co-occurrence of word pairs to examine the difficulties of specific research themes in this area and map them in a two-dimensional space based on their centrality and density.

**Figure 9:**
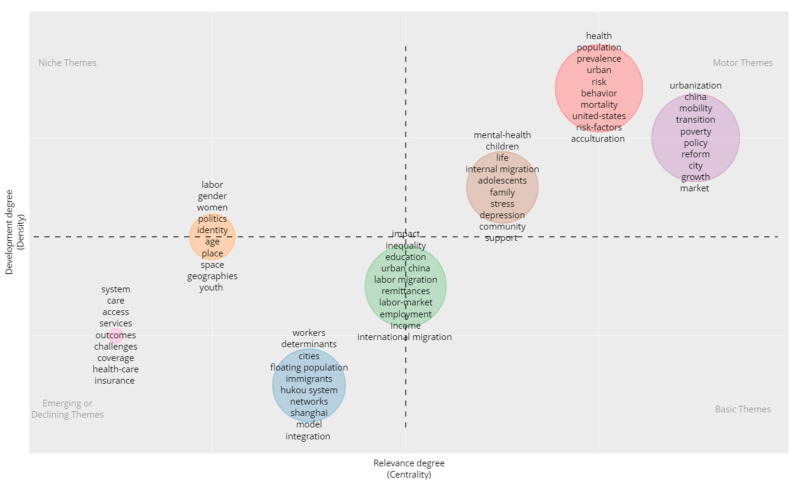
Thematic map of rural-to-urban migrant studies (1 January 2004–3 June 2022).

As indicated in Figure 9, the diagram contains four quadrants: in the first, motor themes are recognized as developed and necessary with high centrality and density (Quadrant I); in the second, niche themes are developed and isolated from other themes (Quadrant II); in the third, emerging or declining themes show low centrality and density, or in other words, they are underdeveloped and marginal (Quadrant III); and in the fourth, basic themes appear that have a low degree of development (Quadrant IV) [53,54]. The themes of this study are mainly found in Quadrants I and III, reflecting two trends: (1) themes specifically related to the labor market are currently receiving less attention, while studies on gender and youth migration may emerge as a niche research field, e.g., [55], and (2) themes related to mental and physical health, as well as the acculturation and social integration of migrants and their family members, form the very essence of migrant studies. We argue that China’s growing academic influence and its unique social structure play an essential role in shifting the center of collective intelligence and guiding both internal and external attention.

#### 3.5.3. Conceptual Structure Map

**Figure 10:**
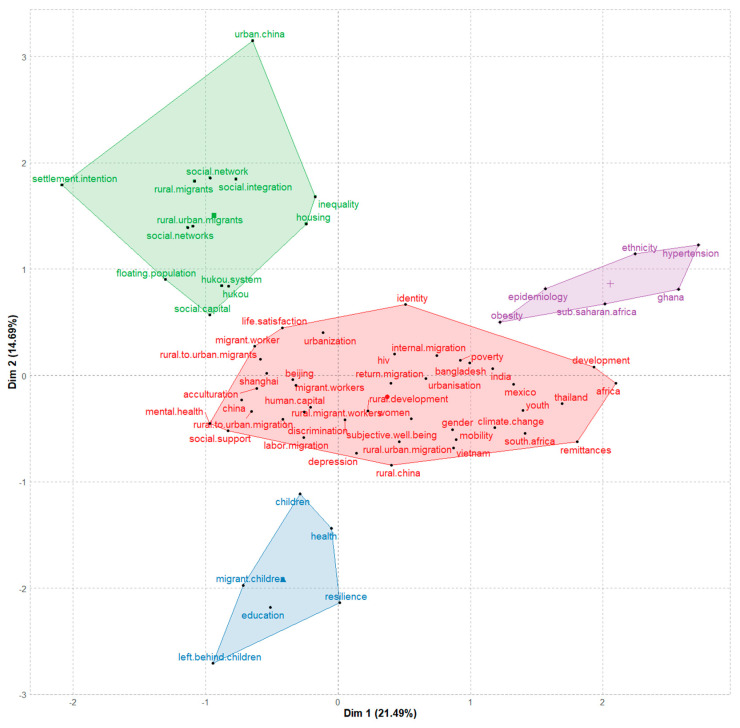
Conceptual structure map of rural-to-urban migrant studies (1 January 2004–3 June 2022).

The multiple correspondence analysis (MCA) method’s built-in R was applied for the generation of a conceptual structure map (Figure 10) [56,57]. We set the minimum occurrences of author keywords to 15. The analysis identified four main clusters: (1) the green cluster, denoting China’s Hukou system and its institutional impact; (2) the red cluster, indicating discrimination and social integration; (3) the blue cluster, which refers to health and well-being; and (4) the purple cluster, centering on epidemiology. The details of each cluster drawn from the documents that contain the highest contributions are provided as follows.

**Green cluster: China’s Hukou system and its institutional impact.** Attention was drawn to the urban development of China as millions of peasants migrated to its metropolises seeking employment and residence. The reasons for their decisions were studied by researchers working from diverse perspectives, such as analyzing the environmental differences between rural and urban localities, e.g., [58,59] and profiling migrants’ demographic characteristics, e.g., [43]. When the Hukou system was established, a boundary was created between China’s urban-dwelling migrants and its native urbanites. The Hukou classification scheme divides China’s population by residential location and socioeconomic eligibility and features a birth-subscribed orientation [37]. These factors have made the conversion of Hukou registration far from a matter of personal choice [36,42,60].

The divide instituted by the state leads to parallel networks of urban rights and distribution of benefits to the urban peasants and their native urban neighbors [61]. Furthermore, a study found that migrant older people have less access to various well-being resources than urban residents due to residential segregation caused by Hukou’s institutional constraints [62]. Concerns about socio-spatial inequalities are echoed in an air pollution study that reveals migrant groups bore a disproportionate share of Beijing’s declining air quality from 2000 to 2010 [63]. As the burden of commuting differs between employees with and without a local Hukou, institutional discrimination arises; the former may exercise their right in terms of accommodation provided by the work unit. Individuals’ housing and employment decisions reflect this inequality [64].

The disparity between the two groups might already have been present in the premarket period due to the unequal educational opportunities available [31]. Despite the increasingly available option of converting to urban Hukou, migrants’ intention to convert their Hukou remains at a low level for several reasons, not least of which is an unwillingness to abandon farmland [65].

Given that the Hukou system has long been China’s central institutional mechanism for shaping the rural-urban and state-society relationships, its complexity was addressed in a range of studies. Topics are covered in multiple areas, including trends and developments, e.g., [41], reforms and changes, e.g., [29,38], and practical conditions and limitations, e.g., [27,66,67].

**Red cluster: Discrimination and social integration.** Findings on occupational segregation and wage differentials between rural migrants and urban residents imply the existence of discrimination against rural groups [40]. Furthermore, policing practices intended to ensure equal justice may lead to the mistreatment of rural migrants in an urban setting [68]. Social discrimination and unfair treatment even impact migrant children, who generally benefit from family unity in the city [69]. Nonetheless, according to Goodburn [70], migrant children in state schools face discrimination from classmates, teachers, and parents of local students. Meanwhile, private schools established by migrant communities face significant strain as a result of tensions between the state and China’s emerging civil society. However, other factors that contribute to the rural–urban divide should not be overlooked, such as “distance from home,” which makes the “digital invisible” circular migrants [71] and “irrational expectations,” which lowers rural–urban migrant households’ subjective well-being [72].

The term “floating population” emphasizes that a certain number of urban migrants consider their adventure in the city to be a temporary strategy, the result of multiple factors, such as the uncertainties of the temporary work labor market [27]. Studies of urban villages, a type of urban neighborhood containing urbanized villagers and migrant workers in various proportions, reinforce the notion that for migrant workers, the city is a workplace rather than a home [35,73]. Circulating between urban work and rural homes is a long-term practice among rural Chinese [32]. Non-kin resident ties (social ties between migrants and local urban residents) play an essential role in community integration [74,75]. In addition to social capital, cultural adaptability and financial resources are crucial for the integration of migrants’ identity [76]. The choice of permanent migration often falls as an optimal combination of various options (distance from home, income stability, etc.) [30].

Generational factors should not be ignored when discussing settlement decisions, as the younger generation of migrants shows greater activity in speeding up their integration into the urban network [34]. Policymakers should recognize newcomers’ needs across a broad spectrum of issues, including housing and social insurance; on the other hand, the role of an urban society, where multiple players such as nongovernment organizations can play their part in social integration [77,78].

**Blue cluster: Health and well-being.** The very nature of a floating labor force raises concerns about health risks to migrants, ranging from occupational disease and injury to infectious diseases and maternal health. Studies also found a positive correlation between high mobility and sexual risk among migrants, as well as an increase in cardiovascular disease, obesity, and diabetes associated with migration into urban areas [79,80,81]. Despite numerous health risks, migrant workers have a lower health insurance rate than the average due to disadvantaged socioeconomic status [82]. Meanwhile, various forms of abuse of female migrant workers can be found across nations [83,84].

Several studies have identified the importance of targeting mental health promotion and mental disorder prevention in the migrant population [28,85]. Taking a broader lens, Wong et al. [26] provide a deeper understanding of rural migrants’ marginalized living experience and its psychosocial impacts on their lives. Attention to issues of mental health and risk perception is needed, due to the complex challenges that migrants are facing, especially younger migrants, known as the second-generation rural migrants, as “youth mining (conscious and unconscious trading of future ill health for present economic opportunities) is a prevalent behavior in migrant populations” [86] (p. 1718). Migration during childhood may contribute to an increased rate of first-episode psychosis [87]. Nevertheless, poor mental health is not necessarily a problem if everyone enjoys an equal likelihood of upward economic mobility, and social capital is relatively high within migrant communities [33].

On the other side, children left behind by one or both of their migrant parents, especially the mother, may suffer disadvantages expressed in their health behavior and school engagement [88,89]. According to studies, school-age children who are left behind experience more victimization and emotional distress, as well as higher anxiety and depression [90,91]. Unsurprisingly, left-behind wives and elders have a lower health-related quality of life [92,93]. Adhikari et al. [94] proposed understanding the differences in healthcare-seeking behavior between the elderly with children present and the elderly left behind to improve the elderly’s physical health.

**Purple cluster: Epidemiology.** Health services are among the most crucial of all the resources designed to help maintain a growing urban population for the success of the system [95]. Migrating to urban areas generally means a better chance of getting healthcare. Yet a link between urbanization and extended epidemics was suggested, where migration might be the trigger [96]. Due to their demographic characteristics of high mobility, lack of education, and low socioeconomic status, migrant groups are considered to be vulnerable to epidemic diseases. During the coronavirus disease 2019 pandemic, Singapore’s lessons highlight the high potential for disease transmission within migrant worker dormitories [97]. At the same time, the increasing populations of rural-to-urban migrants have put a heavy burden on epidemic control (e.g., tuberculosis control) in cities, and migrants’ lack of perception of a need for treatment appears to be a barrier to disease control [98,99]. Apart from the vulnerability of the group itself, the low level of knowledge about epidemics among the general public also contributes to delays in seeking care [100]. However, an increasing number of circular labor migrants who become ill at work and return home to obtain care hints at a need to reconsider the distribution and allocation of healthcare resources [101].

The findings of the study highlight the importance of studying mental illness from an epidemiological standpoint. Saha et al. [102] argued that migrant status is an important risk factor for the increased prevalence of schizophrenia after reviewing 15 migrant studies from eight countries. Moreover, migrant adolescents who struggle to form culturally integrated friendships in school are more likely to have mental health problems [103]. Bhugra et al. [104] emphasized the importance of using epidemiological data to map cultural inconsistency and ethnic density.

Race and ethnicity play a role in epidemiologic studies, not only because people from different racial and ethnic minority groups have different health statuses and health outcomes but also because certain social determinants, such as inequalities and inequities, exist and pose a challenge to the delivery of better public health services [105,106].

As noted, at least four clusters were identified with the MCA method. However, these clusters were not isolated from each other but were entangled from different perspectives. The discussion is open to additional possibilities because the MCA graphics vary with different settings, such as in different keyword stemming fields, and any interpretation of the variables and their interactions must be subject to the author’s limitations.

## 4. Conclusions

This study conducted a bibliometric analysis and had selected results mapped based on 2788 English articles obtained from the WOS Core Collection database. These results provide a rich source of information on the intellectual structure of the chosen domain of rural-to-urban migrants. Using the bibliometrix R-package and VOSviewer, our bibliometric analysis results allowed for network analysis, a document coupling analysis, a reference co-citation analysis, a co-occurrence network analysis, a thematic analysis, and a conceptual structure analysis based on keyword interactions. Efforts were made to promote cross-disciplinary explorations of knowing and understanding the migrant population against the background of fast-growing urbanization.

As China’s unique Hukou system highlights the divide between rural migrants and urban dwellers, migrant studies have extended to a diverse range of interests. We underlined the most productive sources and authors in this area and identified networks of collaboration among countries and institutions among them. Furthermore, we found trends in research themes and topics and research clusters through keyword-based analysis techniques. From disease prevalence as the population moves to labor market dynamics and interactions, to settlement intention and social integration, research trajectories in the migrant research literature are profiled. The ups and downs of specific research themes in this area are examined, and four major cross-disciplinary clusters provide a comprehensive and detailed description of this group of people. The following are some suggestions for future study regarding rural-to-urban migrants:

- It should be noted that population registration systems such as the Hukou system in China are capable of serving multiple interests, that is, they do more than function as an administrative tool to monitor their residents;
- Migrants’ decisions on whether to stay, circulate, or leave the city vary, creating a dynamic environment, and posing challenges for their family and community networks;
- More effort into researching the social determinants of migration and health is required.

Although migration studies may be subject to selection bias due to unobserved characteristics that vary over time and space, a bibliometric analysis may shed light on effective policy interventions to promote migrant livelihood improvement. Policymakers must recognize the value of families and communities in providing a support network for migrants and those who were left behind. Furthermore, occupation-specific medical policies and affordable health insurance are required, as is attention to the digital divide in the information age. Last but not least, potential institutional impacts must be assessed to prevent systemic discrimination and violence against migrant groups.

This study has several limitations. First, further analysis applied to data obtained from multiple sources, such as the Scopus database, could lead to a broader and deeper interpretation of this topic, as we only used data collected from the WOS Core Collection. Second, this study focuses on urban ward migrations, most of which occur in the domestic context, leaving the cross-border population (e.g., immigrants and refugees) out of the picture. Finally, a keyword-based conceptual analysis might only provide a broad but shallow interpretation of the intellectual structure, which could be enhanced by combining it with a systematic review approach.

## Figures and Tables

**Table 1:** Primary information and summary of the dataset.

Description	Results
Timespan	1 January 2004–3 June 2022
Sources	1006
Documents	2788
Average years from publication	6.07
Average citations per document	17.05
Average citations per year per doc	2.123
References	98,860
**DOCUMENT CONTENTS**	
Keywords Plus	3624
Author’s Keywords	5626
**AUTHORS**	
Authors	5761
Author Appearances	9167
Authors of single-authored documents	602
Authors of multi-authored documents	5159
**AUTHORS COLLABORATION**	
Single-authored documents	702
Documents per Author	0.484
Authors per Document	2.07
Co-Authors per Documents	3.29
Collaboration Index	2.47
International co-authorships %	38.2

**Table 2:** Top 10 journals with the most articles published on the topic of rural-to-urban migrants (1 January 2004–3 June 2022).

Journal	JCR Category	N. of Documents	Total Citations
POPULATION SPACE AND PLACE	- DEMOGRAPHY - GEOGRAPHY	65	1171
INTERNATIONAL JOURNAL OF ENVIRONMENTAL RESEARCH AND PUBLIC HEALTH	- PUBLIC, ENVIRONMENTAL and OCCUPATIONAL HEALTH - ENVIRONMENTAL SCIENCES	55	317
CITIES	- URBAN STUDIES	52	908
BMC PUBLIC HEALTH	- PUBLIC, ENVIRONMENTAL and OCCUPATIONAL HEALTH	51	1142
HABITAT INTERNATIONAL	- URBAN STUDIES - DEVELOPMENT STUDIES - REGIONAL and URBAN PLANNING - ENVIRONMENTAL STUDIES	50	2125
PLOS ONE	- MULTIDISCIPLINARY SCIENCES	50	946
CHINA ECONOMIC REVIEW	- ECONOMICS	49	1489
URBAN STUDIES	- URBAN STUDIES - ENVIRONMENTAL STUDIES	48	1420
JOURNAL OF RURAL STUDIES	- REGIONAL and URBAN PLANNING - GEOGRAPHY	40	806
SUSTAINABILITY	- ENVIRONMENTAL STUDIES - GREEN and SUSTAINABLE SCIENCE and TECHNOLOGY - ENVIRONMENTAL SCIENCES - TECHNOLOGY	37	123

**Table 3:** Top 10 countries with the highest number of papers.

Country	Articles	Freq	SCP *	MCP **	MCP Ratio	Total Citations	Citations per Article
CHINA	1035	37.4%	657	378	0.365	14,878	14.37
USA	538	19.5%	328	210	0.390	12,720	23.64
UNITED KINGDOM	241	8.7%	132	109	0.452	5973	24.78
AUSTRALIA	137	5.0%	86	51	0.372	2416	17.64
NETHERLANDS	87	3.1%	35	52	0.598	1480	17.01
INDIA	71	2.6%	59	12	0.169	522	7.35
GERMANY	67	2.4%	46	21	0.313	1175	17.54
CANADA	62	2.2%	34	28	0.452	1171	18.89
SOUTH AFRICA	37	1.3%	27	10	0.270	593	16.03
SINGAPORE	34	1.2%	25	9	0.265	356	10.47
SWEDEN	34	1.2%	23	11	0.324	505	14.85

* SCP: Single Country Publication; ** MCP: Multiple Country Publication.

**Table 4:** Top 10 institutions in the field of rural-to-urban migrants (1 January 2004–3 June 2022).

Affiliations	Country	Articles
Sun Yat-sen University	China	124
Beijing Normal University	China	109
Peking University	China	97
Fudan University	China	83
London School of Hygiene and Tropical Medicine	UK	82
Chinese University of Hong Kong	China	81
University of Hong Kong	China	76
University of Amsterdam	The Netherlands	75
Renmin University of China	China	68
Zhejiang University	China	65

**Table 5:** Top 10 locally cited documents and references on the topic of rural-to-urban migrants (1 January 2004–3 June 2022).

	Cited Journal	LC *
**Most Local Cited Documents**		
Rural migrant workers in urban China: living a marginalized life [26]	International Journal of Social Welfare	123
China’s floating population and their settlement intention in the cities: Beyond the Hukou reform [27]	Habitat International	92
Internal migration and health: re-examining the healthy migrant phenomenon in China [28]	Social Science and Medicine	75
The Household Registration System and Migrant Labor in China: Notes on a Debate [29]	Population and Development Review	71
Circular migration, or permanent stay? Evidence from China’s rural–urban migration [30]	China Economic Review	68
Migrants as second-class workers in urban China? A decomposition analysis [31]	Journal of Comparative Economics	66
Settlement Intention and Split Households: Findings from a Survey of Migrants in Beijing’s Urban Villages [32]	China Review	65
The mental health status of Chinese rural–urban migrant workers [33]	Social Psychiatry and Psychiatric Epidemiology	59
The social networks of new-generation migrants in China’s urbanized villages: A case study of Guangzhou [34]	Habitat International	58
Urban villages under China’s rapid urbanization: Unregulated assets and transitional neighborhoods [35]	Habitat International	57
**Most Local Cited References**		
The Hukou System and Rural-Urban Migration in China: Processes and Changes [36]	China Quarterly	182
Contesting Citizenship in Urban China: Peasant Migrants, the State, and the Logic of the Market [37]	University of California Press	180
Is China Abolishing the Hukou System? [38]	China Quarterly	151
Rural migrant workers in urban China: living a marginalized life [26]	International Journal of Social Welfare	123
Migration, Unemployment and Development: A Two-Sector Analysis [39]	American Economic Review	120
The Two-Tier Labor Market in Urban China: Occupational Segregation and Wage Differentials between Urban Residents and Rural Migrants in Shanghai [40]	Journal of Comparative Economics	115
The Chinese Hukou System at 50 [41]	Eurasian Geography and Economics	110
The Elite, the Natives, and the Outsiders: Migration and Labor Market Segmentation in Urban China [42]	Annals of the American Association of Geographers	101
The settlement intention of China’s floating population in the cities: recent changes and multifaceted individual-level determinants [43]	Population, Space and Place	98
The New Economics of Labor Migration [44]	American Economic Review	96

* LC: local citations.

## Data Availability

The data that support the findings of this study are available from the corresponding author, S. Wang, upon reasonable request.
